# Learning from the GP-consultant exchange scheme: a qualitative evaluation

**DOI:** 10.12688/mep.17542.1

**Published:** 2022-07-11

**Authors:** Pritti Aggarwal, Adam Fraser, Sally Ross, Samantha Scallan

**Affiliations:** 1 Senior Clinical Lead, Hampshire, Southampton and Isle of Wight (HSIoW) Clinical Commissioning Group, Southampton, SO16 4GX, UK; 2 Deputy Director of Primary Medical Care, University of Southampton, Southampton, SO17 1BJ, UK; 3 GP Partner, Living Well Partnership, Southampton, SO19 9GH, UK; 4 GP Partner, Bridges Medical Practice, Weymouth, DT4 7DW, UK; 5 Programme Director at Dorset GP Centre, Bournemouth University, Bournemouth, BH12 5BB, UK; 6 GP Clinical Advisor, NHS England, London, RH6 7DE, UK; 7 Wessex GP Tutor, Appraiser and Sessional GP, HEE Wessex, Winchester, SO21 2RU, UK; 8 GP Education Unit, University Hospitals Southampton, Southampton, SO16 6YD, UK

**Keywords:** Communication, leadership, primary health care, secondary care, empathy, evaluation

## Abstract

Collaborative working across primary and secondary care is crucial to providing high quality patient care. There is still a lack of communication and understanding between primary and secondary care, which can impede collaborative working. The experience of observing colleagues in a different speciality can prompt insight, improve morale and promote collaborative working. The GP-Consultant Exchange Scheme aimed to improve professional understanding, foster deeper partnerships, and ignite opportunities for innovation and/or quality improvement (QI) with co-owned local solutions. This paper gives an overview of how the scheme works and sets out some of the outcomes reported by some 200 Consultants and GPs participants to date. Overall, the participants found the scheme an enjoyable way to reconnect clinicians and allowed them to learn about the challenges faced in different areas within the NHS. This low-cost intervention needs motivated individuals to drive the project forward and make it sustainable, but it can be replicated within any organisation or profession in the NHS.

## Background

The value of observing colleagues in the workplace ( Bridgwood *et al.*, 2018) or sharing experience of different work contexts ( Sampson *et al.*, 2017; Stewart & Cunningham, 2021; Wedderburn *et al.*, 2012) is well recognised in the healthcare workplace exchange literature as promoting insight for doctors located in other clinical contexts, improving communication across medical contexts ( Sampson *et al.*, 2016), and enabling professionals to gain an appreciation of shared aspects of work including challenges and opportunities for development ( Lee *et al*., 2019). Indeed, the value of spending time across clinical contexts is arguably growing in importance for doctors across the continuum of education due to the increasingly interconnected nature of care and appreciation of healthcare matters ( Barata & Rigon, 2015). 

In general practice and family medicine, a number of exchange programmes for established practitioners have been described: Bridgwood *et al.* (2017) describe an evaluation of the Royal College of General Practitioner’s (RCGP) Hippokrates Exchange programme (HEP) which aims to give early career general practitioners experience of primary healthcare in another European country with the aim of developing knowledge and skills, professional development and promoting a global approach to primary care. Zwart *et al.* (2013) describe an international exchange scheme between GP trainers and the Wessex Deanery, UK with educators from University Medical Centre (UMC), Utrecht. Participants described a number of ‘eye openers’ on learning about training in a different context and valued the opportunity to also learn about clinical practice. Van den Heuvel & Hood (2010) describe a military GP trainer exchange which was found to be a useful source of peer-review. Jelley (2002) looked back at ten years of running an exchange for GPs in the UK and Portugal and gathered feedback using interviews. The findings indicated that participants from both contexts valued the opportunity for the insights gained in relation to service provision, teamwork and links. In general practice and family medicine the ‘literature footprint’ reporting structured exchange programmes for established practitioners may be described as sparse; for hospital-based specialties it is hard to find and typically anecdotal e.g. Axon (1998). 

This case study describes the design, management and outcomes of a GP-Consultant Exchange scheme which has been running in the Wessex region for six years ( Ross & Aggarwal, 2019). The purpose of the qualitative evaluation of the scheme was to gather feedback to highlight the benefits of taking part and evaluate the process. It is hoped that readers will find the description useful in sharing the model for wider use.

### Description of the scheme

The first exchange scheme was set up in Portsmouth in 2015 by Dr. Sally Ross. The principles on which the idea was based were to foster trust, respect, mutual understanding, and to improve communication. It was well received, and further exchange programmes have subsequently involved Trusts and GP practices in the localities of Basingstoke, Southampton, Poole, Dorchester, and Bournemouth in more or less the same way. To date, over 200 pairs of consultants and GPs have spent a half day with each other.

Recruitment of participants (consultants and GPs) was via professional networks for example The Local Medical Committee (LMC), Trust staff committees, Wessex Faculty RCGP. Participation in the scheme was by invitation and was voluntary. The scheme was open to consultants and GPs in the relevant localities for each iteration. Those participating undertook a half day exchange programme followed by a ‘celebration meeting’ for participants in the locality. GPs and consultants were paired according to their specialty preference. Participants were then asked to arrange a mutually convenient time to observe each other’s practice for a half-day and, after, to complete a reflection template. Some GPs took their consultant colleague on home visits, some joined team meetings, some spent time with different members of staff in the practice, but most sat in surgery together. The GPs visited a range of hospital departments and experienced acute stroke units, cardiology catheter labs, theatres, outpatient clinics, ward rounds, and medical assessment units. Following the exchange, all participants were invited to share their experiences and learning at a celebratory meeting in their locality. The meetings brought together the clinicians and wider NHS organisations such as the Local Medical Committee (LMC), Trust staff committee, Fourteenfish, Thames Valley Leadership Academy, Heartbeat Charity, Pallant Medical Chambers, and Wessex Faculty RCGP to share learning and outcomes. The meetings varied from area to area for example they might include guest speakers or were recognised and accredited with continuing professional development (CPD) time (see Ross & Aggarwal (2019) for a guide). By way of illustration, an iteration of the scheme typically took 5 to 6 months, including engagement (1 month), promotion, pairing /matching (1–2 months), exchange visits (2–3 months) and a celebratory meeting. Each iteration of the scheme ran when there was enough interest in a locality to sustain a group. 

## Evaluation

### Evaluation design

The purpose of the evaluation of the scheme was to gather feedback to highlight the benefits of taking part and evaluate the process. A questionnaire was used that included a mix of scaled and free text questions. This was completed across all scheme localities between January and September 2019.

### Sampling population

The sampling strategy for the evaluation was to invite all past participants in the scheme to provide feedback. Participation was voluntary. The purpose of the qualitative evaluation of the scheme was to gather feedback to highlight the benefits of taking part, the process and share the model for wider use. Thus, in analysing the evaluative data the focus was on breadth and variety of responses rather than saturation as might be found in research.

### Data collection

Follow up evaluative data was gathered using an anonymous, online questionnaire created on the on the Survey Monkey™ platform which was distributed across all scheme localities over a 9-month period between January and September 2019 (a copy of the survey used can be found under *Extended data*). These were Portsmouth, Basingstoke, Southampton, Poole, Dorchester and Bournemouth which are all within Health Education England (Wessex). For the scheme evaluation, participation was invited and extended to the GPs and consultants who had participated in the scheme across all the localities. Two hundred former exchange participants were sent an electronic survey to complete anonymously in order to capture their experiences and thoughts on the exchange scheme.

Respondents were asked to quantitively rate their experience on a six-point Likert Scale from one (least useful/likely) to six (most useful/likely). Respondents were also asked three open questions about the exchange:

- Did anything surprise you or shock you during the visit?
- What did you expect to see, that you didn’t see? (Or, what did you see, that you weren’t expecting?)
- What will you take back to your own place of work or clinical practice, as a result of this experience?

### Data analysis

The free text responses were qualitatively analysed by topic/theme content following Saldaña (2009), and by participant group, i.e. consultants or GPs. Of particular interest were comments related to the benefit of participation and feedback on the process. This data was then placed in the context of the discussions captured during the celebratory events by the scheme organisers to distil the learning to inform the next iteration. Responses were received from 75 scheme participants, including 34 consultants and 41 GPs. This gave a response rate of 37.5%.

## Ethics and consent

Formal ethical approval was not sought as the authors did not have access to a formal ethics review committee. The evaluation was conducted in accordance with the Declaration of Helsinki. The feedback data were non-sensitive in nature and gathered anonymously, and those providing feedback were informed that the information would be used to evaluate the scheme and may include verbatim quotes. They consented to this use only when providing their feedback. 

## Results

**Figure 1.**
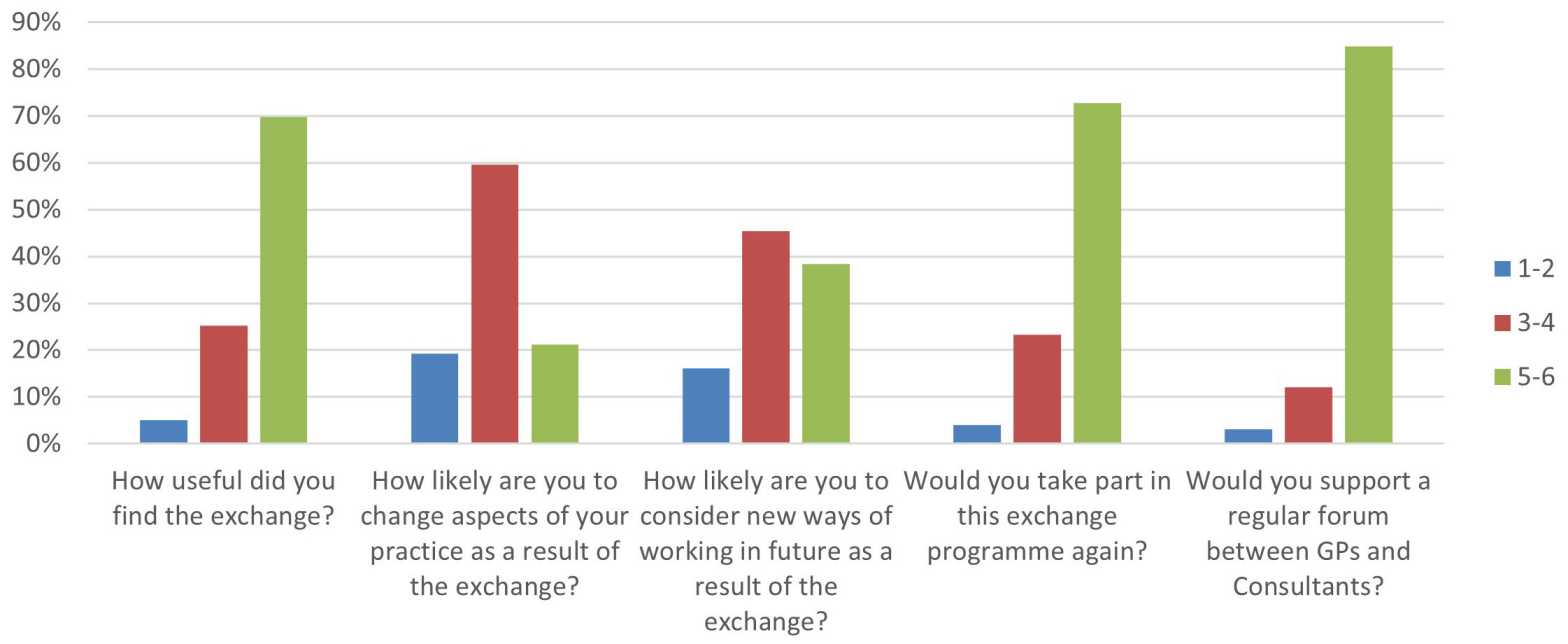
The percentage of individuals who responded negatively (1–2, blue column), medially (3–4, red column) and positively (5–6, green column) out of a maximum of 6 points for each question.

The majority of respondents (70%) found the exchange useful (mean score 4.59). 73% of respondents said they would take part in an exchange again (mean score 4.83) and 85% would support regular exchange forums (mean score 5.25). When asked whether the exchange would change aspects of their practice or encourage new ways of working, the impact was less clear (see Figure 1).

Looking at the free-text responses, participants felt that they had learned from taking part in the scheme. It was found to improve understanding between colleagues by challenging stereotypical views and generating goodwill. There was a strong sense that the experience of observing a colleague in a different specialty rekindled a sense of collegiality. Table 1 sets out the GPs’ reflections of spending time with consultants. Table 2 sets out the consultants’ reflections of spending time with GPs.

**Table 1.** GPs’ reflections on secondary care.

GP’s reflections on secondary care
*Teamworking* “How much teamwork there is in secondary care.” “The amazing camaraderie in a very difficult working environment that provides support for the staff.” *Cases* “It was interesting to see the case mix that the neurologist saw, and how much of it was not specific to neurology but more general – e.g. chronic fatigue /anxiety etc.” “I joined a geriatrics consultant and saw the ongoing pressures on community care from the other side. I perhaps was a little surprised at the level of involvement the geris team still have after discharge that I wasn’t previously aware of.” *Resources* “I was really impressed by the efforts the consultant had gone to develop training materials for doctors and patients. Quite inspirational.” *Infrastructure differences* “I was surprised that the consultant only had a hospital script pad and not able to do NHS script for normal chemist. This meant that patients seen after the pharmacy had closed, or if medication not in stock, the patient would need to travel back to hospital to pick up script and some patients lived far away.” “I witnessed several examples of the everyday frustrations and barriers secondary care clinicians encounter due to poor interfaces between primary and secondary care. I hadn’t expected to see as much of this.” “It was interesting how keen he was to see the XXX cardiology services from a GP’s perspective. It seems a shame that the current commissioning structure doesn’t seem to allow this sort of informal, friendly discussion to shape services more.” *Complexity* “Whilst attending a cancer MDT I witnessed how secondary care consultants despite being specialists have to manage a lot of uncertainty in the diagnosis and management of some of their more complex patients.” “In the main we saw some very complicated diabetic patients, across the hospital. Clearly ward based clinicians have been well trained to manage diabetes without specialist intervention.” *Learning* “I wish we had more TIME in general practice to be able to do this too - it would be so much more satisfying for patients and for myself.” “Appreciate how hard our consultant colleagues work. I also realised that they don’t have the luxury of having the patients whole medical record or drug history in front of them, hence the importance of giving as much details, in the referral letters we send. I can appreciate why they send the proformas for us to prescribe medications that they recommend.” “The value of knowing our colleagues personally; realising we’re in it together.” “Perhaps the most surprising thing was how many of the same issues we are both dealing with. It was clear to me that knowing and understanding the specialists that I refer to has tremendous benefits for the patients we serve and, ultimately, can improve the care they receive.” “How similar our work really is and that we are all trying to do the best for our patients.” “Shocked on the size of the consultant offices they look more like cupboards shared between 2 people!”

**Table 2.** Consultants’ reflections on primary care.

Consultant’s reflections on primary care
*Teamworking* “Team working in the practice really was apparent and they appeared very close knit.” “Although teamwork was apparently taking place via a group messenger application on the doctor’s desktop, I felt they mostly practiced in isolation during the bulk of their working day. However, I still came away with a sense that the group have a strong vision and they work cohesively.” *Cases* “Given the nature of the role, the caseload was varied and largely unpredictable. There was a lot more mental health and illicit drug related issues than I tend to encounter with inpatient work, which can be challenging.” “The efficiency of GP to navigate through complex case and medications in short time.” “The competence in dealing with such a wide range of patients.” “We went on a home visit to see a patient – if I’d seen her in clinic I suspect I wouldn’t have had any idea that she was a hoarder who lived in total chaos … Similarly, we saw two regular patients who have incredibly difficult personal circumstances, including abusive relationships and benzodiazepine dependency etc. that might well be missed by a hospital team.” “The obvious lack of awareness of the reality of the pressures in general practice and the variety of the patient presentation…. Also my ignorance of the skill set within general practice team.” *Resources* “Impressed at how technology has been embraced in the community but the amount of admin done is staggering.” *Infrastructure differences* “I also didn’t realise how the GP partners were involved in shaping primary care in their area.” *Complexity* “I was not surprised but impressed by the competence in dealing with a very wide range of patient types (from a tiny baby to an elderly gentleman). The patients were probably slightly more complex as a whole than might have been expected and several needed further input after the consultation (referral or telephone calls for advice).” *Learning* “GPs need to sell themselves as specialist generalists more!” “Level of isolation of GP’s and responsibility – the buck literally stops with them!” “Listening style – I tend to write whilst listening. It was good to see another clinician’s communication style. My GP was an active listener. We often don’t know the answer to a problem – be confident in your uncertainty.” “That some GP’s really go the extra mile with their patients (setting up a support group for women suffering from domestic violence).” “I already had a great respect for my GP colleagues which has been strengthened. I think their job is really difficult.” “Respect – when GPs call in they are calling on their clinical experience and are more often right than wrong about a diagnosis.” “A better sense of how I can support general practitioners with their queries and managing liver patients in the community. I learnt some secondary care consultation letters were more helpful than others. As an example, we perform a test (Fibroscan) which provides a value on how stiff the liver is, when interpreted it allows a diagnosis of liver fibrosis to be made. We have improved our written communication to the GPs to provide an interpretation rather than just the raw value.”

Participants felt that the exchange was a useful educational experience and proposed that it should be a mandatory part of training.

*“A great insight after 23 years of hospital practice.”* [Consultant]
“ *I was really impressed by the efforts the consultant had gone to develop training materials for doctors and patients. Quite inspirational*.” [GP]
*“I will encourage colleagues to consider it and I will suggest to training committee that we ask specialist registrars to do a day a week each year in general practice.”* [Consultant]
*“How much team work there is in secondary care”* [GP]
*“Consider sending every SpR out to primary care for a week before they become a consultant – would be really good for them especially if they have not done primary care.”* [Consultant]
*“It was interesting to see the case mix that the neurologist saw, and how much of it was not specific to neurology but more general – e.g. chronic fatigue/anxiety etc.”* [GP]
*“I was not surprised but impressed by the competence in dealing with a very wide range of patient types (from a tiny baby to an elderly gentleman). The patients were probably slightly more complex as a whole than might have been expected and several needed further input after the consultation (referral or telephone calls for advice).”* [Consultant]
*“I joined a geriatrics consultant and saw the ongoing pressures on community care from the other side. I perhaps was a little surprised at the level of involvement the geris* [geriatrics] *team still have after discharge that I wasn’t previously aware of.”* [GP]

Time spent in each other’s environments enabled participants to appreciate the challenges they each faced within the NHS. This seemed to generate greater mutual respect that led to strengthened professional relationships, and a shared understanding of working. Participants reflected on the importance of teamwork within the clinical environment. There was recognition that collaborative working was a necessity in the modern health service due to increasing service pressures. Many participants were impressed by their exchange partner’s practice, which may have been more apparent because doctors observed a specialty in which they had little expertise themselves. Many participants felt increased respect for each other’s ability to manage considerable complexity and uncertainty. Consultants highlighted the complexity in primary care of medical decision-making, in particular managing risk and uncertainty. GPs recognised that patients in hospital have become more complex as people live longer with more co-morbidities. There were comments indicating that consultants have access to more investigations, which may help to manage risk. It was also recognised that investigations may sometimes provide false reassurance. Where challenges were identified these concerned the difficulties of communication across the primary-secondary care boundary.

In a small number of cases the exchange led to tangible quality improvement activity. Two examples which were identified in the feedback concerned:

1. Dr. G spent a morning in general practice and observed how many templates were being used to code and structure the consultation. Dr. G reflected on the experience and consequently has piloted a template structure for their outpatient department (OPD) letters. The benefit of this has been seen in terms of time efficiency as each letter could take up to 30 minutes to dictate. Having a template structure guides junior trainees in structuring their own letters and promotes consistency.
2. Dr. P spent time in general practice and reflected that 90% of the letters were being read by the administration team in primary care. They coded letters and only passed ones on to GPs which required the GP to action something specific. Dr. P considered this and has begun to change their own clinical practice by giving letters a heading, for example ‘For Information Only’ or ‘GP Action required.’ Having identified that doing this was helpful to the administration team in primary care, the step was taken to request that all specialties head letters with ‘GP information only’ / ‘GP Action required.’

## Discussion

The aim of this qualitative evaluation has been to present feedback to highlight the benefits of taking part in the GP-Consultant Exchange Scheme. Past participants in the scheme valued the time spent across clinical contexts as it provided insights into the clinical practice and context of others. These findings echo those of other exchange schemes for established clinicians whether national or international. Such time is valuable; it can develop clinical practice ( Axon, 1998) and enhance appreciation of care and clinical practice in different contexts ( Bridgwood *et al.*, 2017; Bridgwood *et al.*, 2018; Jelley, 2002; Van Dormael *et al.*, 2007; Van den Heuvel & Hood (2010); Zwart *et al.,* 2013).

### Implications and future developments

Whilst the exchange scheme is straightforward to implement and cost-effective, it needs an enthusiastic individual to drive the set up and matching process and provide leadership. Identifying a ‘champion’ in the organisations involved was also found to facilitate the process.

A few factors need careful consideration before embarking on running a scheme. These are:

1. *Time:* Non-participants cited time as a significant issue in implementing the exchange. Time of the year would need further thought particularly in primary care, as the last quarter of the financial year was a particularly difficult period to host a consultant. Allocated time and administration support for the organisers are key factors that maintained drive.
2. *Funding:* Consultants were encouraged to use structured programme activity (SPA) time, whereas GPs had to do their reciprocal visits in their own time, thus all schemes in Wessex were unfunded for GPs. Those that participated could see the benefits from the onset. Organisers did have some GPs complaints about the hospital parking.
3. *Fear:* There is a fear of being observed by a colleague. This was perceived much more in primary care where GPs are particularly isolated and used to working by themselves in a room with a patient. Some attitudes of ‘I have nothing to gain or learn from this opportunity’ were also experienced by several of the organisers.

The GP-Consultant Exchange Scheme was a simple, low-cost intervention that demonstrated an impact on participants. The exchanges provided opportunities for relationships and building trust, which are crucial to developing a mutual understanding of the challenges we all face. The scheme lends itself to wider use within any NHS organisation or professional group, for example:

- It can allow trainees to consider the wider healthcare system during their training,
- It can increase healthcare professionals’ knowledge of the primary secondary interface,
- It can support continuous professional development by considering the patient experience with fresh eyes.

It does, however, need motivated and tenacious individuals to drive the project forward for the benefit of the local system and participants.

### Take home messages

1. The GP-consultant exchange scheme is an enjoyable low-cost quality improvement activity.
2. The scheme can be replicated in any setting.
3. The GP-consultant exchange scheme provides space and time for mutual understandings of the challenge’s primary and secondary care face in the current NHS.
4. Through mutual appreciation local solutions and learning has supported better patient care.
5. It requires a motivated individual to champion the project and tease out the learning opportunities for the benefit of the system and participants.

## Data availability

The underlying data to this case study cannot be shared as participants did not consent to it being used in this way and/or being made available in a repository. The evaluation section contains a description of the data gathered and how it was gathered to allow replication of the study. Any queries about the method and/or data should be directed to the corresponding author.

### Extended data

OSF: [GP-consultant exchange scheme]. https://doi.org/10.17605/OSF.IO/9Z4QG ( Aggarwal *et al.,* 2022)

This project contains the following underlying data:

- Data tables.pdf

This project contains the following extended data:

- SurveyMonkey_evaluation_questions.pdf

Data are available under the terms of the Creative Commons Attribution 4.0 International license (CC-BY 4.0).
