# Supplementary material for: Learning from the GP-consultant exchange scheme: a qualitative evaluation
Source: MedEdPublish (2016). 2022 Jul 11;12:51. [Version 1] doi: 10.12688/mep.17542.1 (PMC9427078; doi:10.12688/mep.17542.1)
Supplement: Peer review history

# Peer review: Reviewer response for version 1

**DOI:** 10.21956/mep.18817.r32327

**Published:** 2022-08-08

**Authors:** Peter Cantillon

**Affiliations:** 1 Discipline of General Practice, National University of Ireland, Galway, Ireland

This is an interesting evaluation of a general practice/consultant exchange scheme operated for several years in the Wessex region of England. Using a mixed cross-sectional quantitative and qualitative survey design the authors have provided very interesting insights into the benefits and some of the barriers to such schemes. To my knowledge, this is one of very few such evaluative studies and thus is an important addition to the literature. I have a few comments that might, I hope, be helpful for the authors.

 Whilst this is not a research paper, I would have welcomed an initial review of the problems in primary secondary care communication/interaction that this initiative sets out to address and ameliorate. Thus, rather than reviewing what is already known about placing general practitioners in other countries or other settings, I would have preferred a stronger sense of the rationale for engaging in consultant/GP swap initiatives.

 I think that the survey approach employed in this evaluation was very appropriate, but I'm not sure how the evaluative instrument was designed nor how it was interpreted by participants. It might be helpful to provide some data on this even though, it is not a research study.

 The citations are all very powerful and very appropriate – I wasn't clear however why there were two tables of citations and then further citations listed in the text. I wonder whether it might be preferable to either use the table format or to weave a smaller selection of carefully selected citations into the text to make the point that the authors want to make. I found myself learning somewhat different things from the textual description of what participants have been saying than what I was reading into the citations – linking the citations directly to the interpretations might help to ensure a greater sense of the inferences being drawn.

 Was there any opportunity to examine the opportunity costs or actual costs in terms of time incurred by consultants and general practitioners in this initiative? Having done something similar in the 1990s in London with Dr Hilary Lavender I can remember that taking time out of clinics to do this was one of the main hindrances. It would be good to get a sense of the actual temporal or financial cost.

 In short, this is valuable work with some excellent data extracted from what appears to have been an effective evaluative study. I do hope that the above comments are helpful.

Is the case presented with sufficient detail to be useful for teaching or other practitioners?

Yes

Is the work clearly and accurately presented and does it cite the current literature?

Yes

If applicable, is the statistical analysis and its interpretation appropriate?

Yes

Is the case’s background and context in sufficient detail?

Yes

Are all the source data underlying the results available to ensure full reproducibility?

Partly

Are the conclusions drawn adequately supported by the results?

Yes

Reviewer Expertise:

Clinical Education and Workplace Learning

I confirm that I have read this submission and believe that I have an appropriate level of expertise to confirm that it is of an acceptable scientific standard.

# Peer review: Reviewer response for version 1

**DOI:** 10.21956/mep.18817.r32330

**Published:** 2022-07-26

**Authors:** Louise Dubras

**Affiliations:** 1 School of Medicine, Ulster University, Coleraine, UK

There is little existing literature regarding exchange schemes between GPs and hospital consultants, so the authors are to be congratulated on writing up this work. The description of the workings of the scheme is clear, which should provide sufficient information for others keen to consider running a similar scheme. In the description of the scheme there is reference to organizations by name: These might not be familiar to readers so it would be wise to explain the function of the organizations rather than give their names. 

 There are some areas where writing could be more clear: for example the sentence, *"The purpose of the evaluation of the scheme was to gather feedback to highlight the benefits of taking part and evaluate the process" *seemed somewhat cumbersome. 

 I would encourage a slightly more balanced approach to the conclusions. The scheme was run on a shoestring, and it is clear that whilst participants benefited from it, money was a barrier for GPs in particular, as they did this in their own time. Therefore were the participants those who were most keen and most committed? A more critical evaluation might address whether there should be more attention paid to the costs of such a scheme ( or even a cost- benefit analysis). Was this sample of highly committed GPs representative? What about those who did not participate? Why didn't they? 

 I would like to see the authors consider following the work up.

Is the case presented with sufficient detail to be useful for teaching or other practitioners?

Yes

Is the work clearly and accurately presented and does it cite the current literature?

Partly

If applicable, is the statistical analysis and its interpretation appropriate?

Not applicable

Is the case’s background and context in sufficient detail?

Yes

Are all the source data underlying the results available to ensure full reproducibility?

Partly

Are the conclusions drawn adequately supported by the results?

Yes

Reviewer Expertise:

Medical education

I confirm that I have read this submission and believe that I have an appropriate level of expertise to confirm that it is of an acceptable scientific standard.

# Peer review: Reviewer response for version 1

**DOI:** 10.21956/mep.18817.r32329

**Published:** 2022-07-21

**Authors:** Peter Hockey

**Affiliations:** 1 Research and Education Network, Western Sydney Local Health District, Sydney, NSW, Australia; 2 University of Sydney, Sydney, NSW, Australia

A useful Case Study with broader applicability to other health systems. Some specific comments:

- I'm not sure that not having access to a formal ethics review committee is correct. Authors have affiliations with both the University of Southampton and University Hospitals Southampton both of which have ethics committees. Perhaps the study should be better described as a quality assurance process or an evaluation and state that Ethics approval was not required?
- I would not describe this as a low-cost intervention as stated. It is in fact high-cost when individual's time is accounted for and opportunity costs are taken into account. This should be acknowledged and would be reasonable to state that little up-front investment is required.
- The discussion could be strengthened as I believe the reflections demonstrate enhanced mutual respect between primary and secondary care, an appreciation for the value of generalism in medical practice and a number of similarities in ways of working.
- I would strengthen the discussion by stating that this sort of intervention, particularly after the ravages of COVID, can help build collegiality and teamworking so needed in modern-day medical practice.
- There are a number of inappropriately used apostrophes.

Is the case presented with sufficient detail to be useful for teaching or other practitioners?

Yes

Is the work clearly and accurately presented and does it cite the current literature?

Yes

If applicable, is the statistical analysis and its interpretation appropriate?

Not applicable

Is the case’s background and context in sufficient detail?

Yes

Are all the source data underlying the results available to ensure full reproducibility?

No

Are the conclusions drawn adequately supported by the results?

Partly

Reviewer Expertise:

Systems improvement

I confirm that I have read this submission and believe that I have an appropriate level of expertise to confirm that it is of an acceptable scientific standard.
